# Transcriptome analysis and identification of P450 genes relevant to imidacloprid detoxification in *Bradysia odoriphaga*

**DOI:** 10.1038/s41598-018-20981-2

**Published:** 2018-02-07

**Authors:** Chengyu Chen, Cuicui Wang, Ying Liu, Xueyan Shi, Xiwu Gao

**Affiliations:** Department of Entomology, College of Plant Protection, China Agricultural University, Beijing, 100193 China

## Abstract

Pesticide tolerance poses many challenges for pest control, particularly for destructive pests such as *Bradysia odoriphaga*. Imidacloprid has been used to control *B. odoriphaga* since 2013, however, imidacloprid resistance in *B. odoriphaga* has developed in recent years. Identifying actual and potential genes involved in detoxification metabolism of imidacloprid could offer solutions for controlling this insect. In this study, RNA-seq was used to explore differentially expressed genes in *B. odoriphaga* that respond to imidacloprid treatment. Differential expression data between imidacloprid treatment and the control revealed 281 transcripts (176 with annotations) showing upregulation and 394 transcripts (235 with annotations) showing downregulation. Among them, differential expression levels of seven P450 unigenes were associated with imidacloprid detoxification mechanism, with 4 unigenes that were upregulated and 3 unigenes that were downregulated. The qRT-PCR results of the seven differential expression P450 unigenes after imidacloprid treatment were consistent with RNA-Seq data. Furthermore, oral delivery mediated RNA interference of these four upregulated P450 unigenes followed by an insecticide bioassay significantly increased the mortality of imidacloprid-treated *B. odoriphaga*. This result indicated that the four upregulated P450s are involved in detoxification of imidacloprid. This study provides a genetic basis for further exploring P450 genes for imidacloprid detoxification in *B. odoriphaga*.

## Introduction

The sciarid fly *Bradysia odoriphaga* is a serious crop pest with a wide range of more than 30 plant species from seven families^1,2^. The main host plant of *B. odoriphaga* is Chinese chive (*Allium tuberosum* Rottle ex Spreng). Chinese chive is a perennial vegetable with a high economic value and is grown over a vast geographic area from Asia through the Middle East, to Europe and North America, and is widely cultivated in China^3–7^. *B. odoriphaga* larvae usually gather in the roots, bulbs, and even in immature stems of Chinese chives, making the pest hard to control and allowing it to cause significant production losses of Chinese chives^8^. Failing to control *B. odoriphaga* could cause 50% yield reduction in Chinese chives at harvest time^9,10^.

Among the commonly used insecticides for control of *B. odoriphaga* larvae, neonicotinoids such as imidacloprid exhibit a good control efficacy^11,12^. Since 2013, insecticides containing imidacloprid as active ingredient has accounted for more than 60% of insecticides registered with the Ministry of Agriculture in China for *B. odoriphaga* control (www.chinapesticide.gov.cn). With the wide application of imidacloprid for *B. odoriphaga* control, increased imidacloprid resistance has recently developed in field populations of *B. odoriphaga*^13–15^. Therefore, in order to delay imidacloprid resistance development in *B. odoriphaga*, it is crucial to identify the imidacloprid detoxification metabolism related genes in *B. odoriphaga*, and then figure out the mechanism of imidacloprid detoxification.

The pathways by which imidacloprid is metabolized and detoxified in insects have been reported widely, highlighting the principal roles of cytochrome P450 enzyme and elevated gene expression levels of P450s in imidacloprid detoxification^16,17^. P450s are a superfamily of enzymes with many functions, including nutrition and xenobiotic detoxification by metabolizing a wide range of endogenous and exogenous compounds^18,19^. The over-expression of some P450 genes has been proven to be associated with increased metabolism of imidacloprid, including *CYP6G1* (*Drosophila melanogaster*), *CYP6D1* (*Musca domestica*), *CYP6ER1, CYP6AY1* (*Nilaparvata lugens*), and *CYP6CM1vQ* (*Bemisia tabaci*)^20–23^. Therefore, the over-expression of P450s is a common mechanism to detoxify imidacloprid in insects. In addition, elevated P450s enzyme activity was found to be related with imidacloprid insensitivity in *B. odoriphaga* larvae in our previous study, which is currently the only report about the imidacloprid detoxification mechanism in *B. odoriphaga*^14^.

Although many ecological and physiological aspects of *B. odoriphaga* have been investigated in recent years^1,24,25^, the molecular mechanisms for insecticide metabolism in *B. odoriphaga* are still unexplored, especially those mechanisms related to metabolism of the commonly used insecticide imidacloprid. Recently, next-generation sequencing (NGS)-based RNA-Seq analysis has enhanced the efficiency and speed at which genes are discovered, especially for insects without a reference genome^26^. Moreover, transcriptome sequencing is an efficient way to uncover information about functional genes and differentially expressed genes in insects after insecticide treatment^27,28^.

In this study, we generated annotated transcriptome sequences and gene expression profiles that provide useful information for identifying genes involved in imidacloprid detoxification in *B. odoriphaga*. The assembled and annotated unigenes in the transcriptome were selected to explore key P450 genes related to imidacloprid detoxification, and to provide a molecular basis for exploring imidacloprid detoxification and functional analysis of these genes. In addition, the gene function of upregulated P450 transcripts were further explored through RNAi, which provided a better understanding of the role of these differentially expressed P450 genes in imidacloprid detoxification.

## Results

### Illumina sequencing and *de novo* assembly

According to the results of the bioassay, the LC_50_ value of imidacloprid against *B. odoriphaga* was 4.08 mg/L. Illumina sequencing and *de novo* assembly were performed by merging all samples of *B. odoriphaga* in the treatment and control groups, resulting in the generation of 43,453 total unigenes, a total length of 55,884,005 bp, an N50 length of 1,995 bp, and a mean length of 1,286.08 bp. The corresponding information of transcripts of *B. odoriphaga* is shown in Tables 1 and 2. The size distribution indicated that the lengths of 17,952 of the unigenes were more than 1000 bp (Fig. S1). The control 2 library produced the most data (52,347,280 clean reads), while the control 1 library produced the fewest clean reads (50,957,216). As a whole, all libraries exhibited good quality, with an average of 89.04% of the clean reads meeting base call quality at the Q30 standard. According to the alignments of sequences in the unigene library, the statistics of mapped reads in each sample are shown in Table 2. These clean reads were stored in the NCBI SRA database (http://www.ncbi.nlm.nih.gov/sra/) under the accession numbers SRX2939421.

**Table 1:** Statistics for assembled unigenes.

	Total number	N50 length	Total Length	Max Length	Min Length	Average Length
Unigene	43453	1995	55884005	27155	301	1286.08

**Table 2:** Aligning statistics of clean reads with assembled unigenes.

Sample	Clean reads	Clean nucleotides (bp)	>Q30 (%)	(G + C) %	Mapped reads	Mapped ratio
Control 1	50957216	6369559907	87.93	41.50	45343685	88.98
Control 2	52347280	6543269039	89.67	42.00	46485565	88.80
Treatment group 1	51477692	6434711500	88.12	41.00	45542976	88.47
Treatment group 2	52326200	6540775000	90.43	42.00	46503068	88.87
All	207108388	25888315446				
mean			89.04	41.63	45968824	88.78

### Functional annotation of all unigenes

To classify the functions of predicted unigenes from the imidacloprid treatment and control groups, 21,705 of 43,453 total unigenes in *B. odoriphaga* were annotated using the NR (19442), SWISSPROT (16094), KOG (14723), KEGG (6737), and GO (15245) databases with a cut-off E-value of 10^−5^. For NR annotation, 44.74% of all unigenes provided a BLAST result, and the best-match results of the NR homologous species distribution are shown in Fig. S2. The sequences of *B. odoriphaga* showed 1820 matches with *Aedes aegypti* sequences followed by *Vitrella brassicaformis* (1394), *Ae. albopictus* (1147), and *Culex quinquefasciatus* (1143) (Fig. S2). Gene ontology (GO) analysis gives the representation of gene and gene product attributes across all libraries. In each of the three main categories (biological process, cellular component, and molecular function) of the GO classification, the terms “binding”, “cell part”, and “cellular process” were the most dominant, respectively (Fig. S3).

### Expression variation of differential expression genes (DEG) related to imidacloprid detoxification

**Figure 1:**
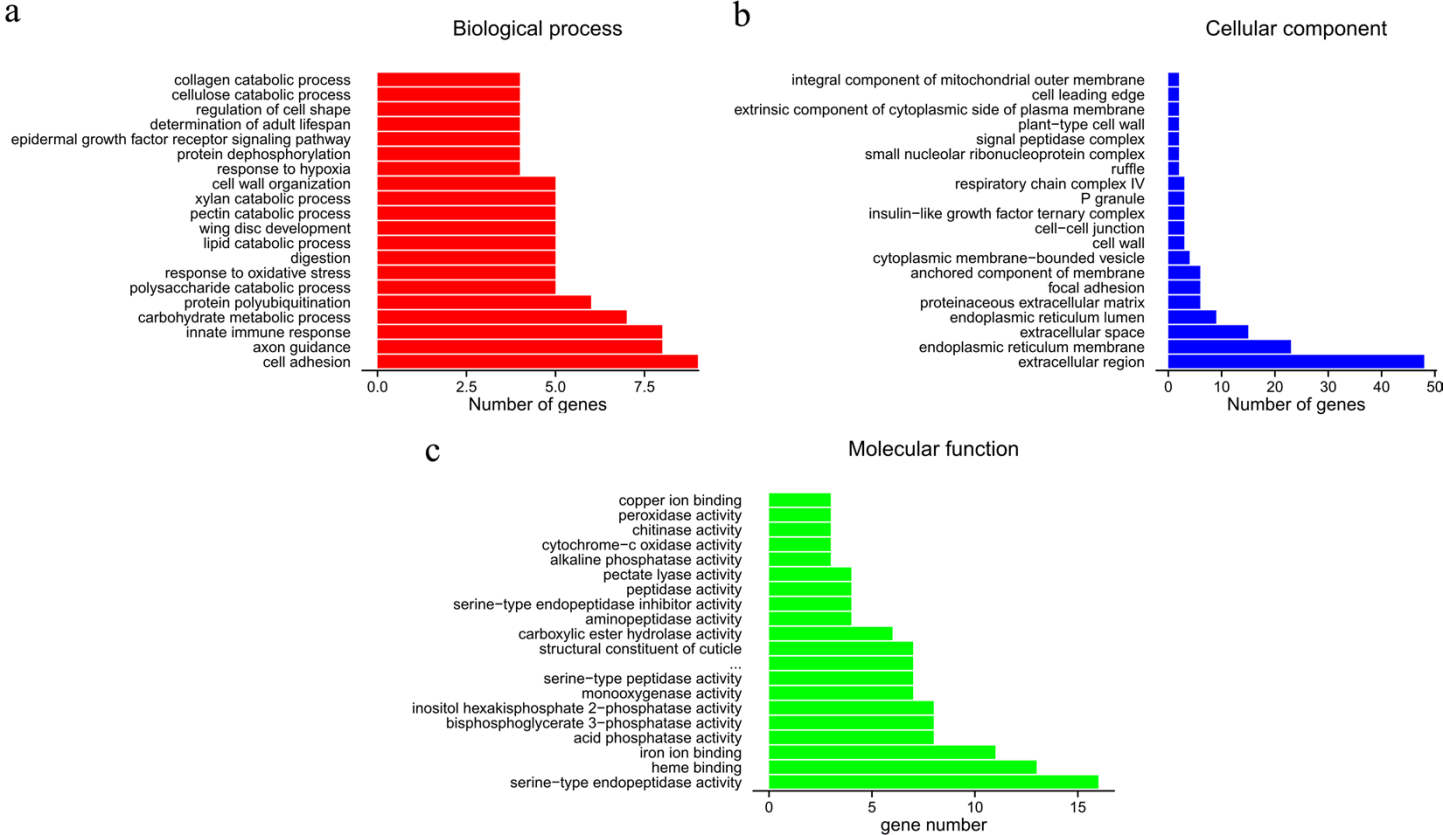
The distribution of differential expression genes (DEGs) annotated in the Gene Ontology (GO) data library within the biological process (**a**), cellular component (**b**), and molecular function (**c**) categories. The x-axis indicates the number of unigenes sub-categories and the y-axis indicates the sub-categories.

**Figure 2:**
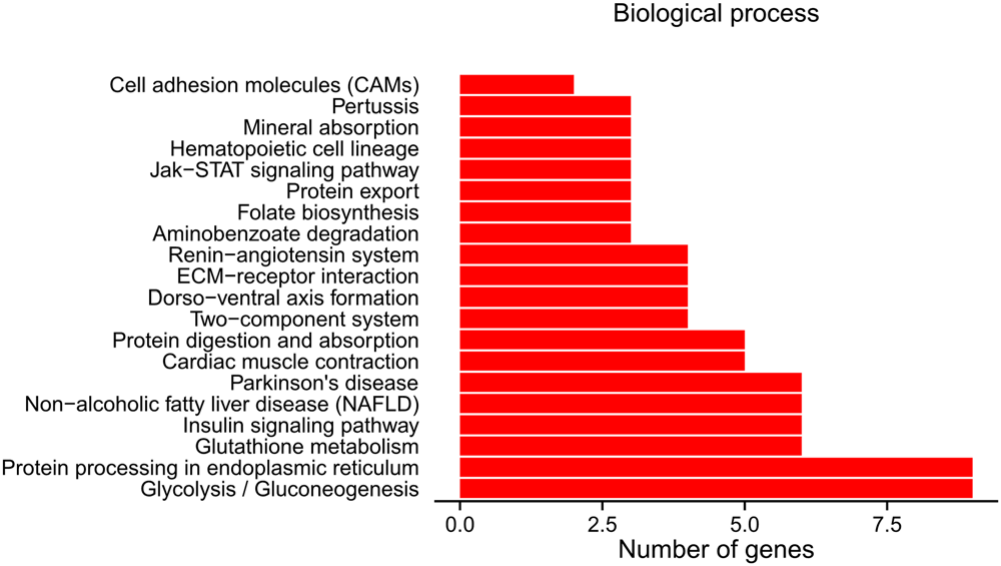
The distribution of pathways of differential expression genes (DEGs) annotated in the Kyoto Encyclopedia of Genes and Genomes (KEGG) data library. The x-axis indicates the number of unigenes sub-categories and the y-axis indicates the sub-categories.

Based on the DEG analysis, out of 674 DEGs between imidacloprid treatment and control, 281 and 393 unigenes were upregulated and downregulated, respectively (Figs 1 and 2).

In the comparison of imidacloprid treated and control larvae, 360 DEGs were annotated in the NR database. Among them, seven P450 unigenes, which are named with comp 30097, 30167, 34369, 36247, 38558, 40700 and 44013, were obtained according to the standard described above (Table 3). There are four P450 unigenes were upregulated (comp 30167, 38558, 40700 and 44013), while the other three P450 unigenes were downregulated (comp 30097, 34369 and 36247). Among the three main detoxifying enzymes in insect, namely P450s, carboxylesterase and GSTs, besides P450s, we also found a carboxylesterase unigene, but it showed down regulated. Thus, we selected these seven P450 unigenes as candidates to evaluate gene expression profiles.

**Table 3:** The imidacloprid detoxification related P450s genes detected in the *Bradysia odoriphaga* unigenes dataset.

Unigene ID	Accession ID	NR annotation	RNA-seq	
			Log_2_ FC	*P* value
comp30167	gi|170049288	*cytochrome P450 9b2*	1.179	0.016
comp38558	gi|568255592	*cytochrome P450*	0.398	0.033
comp40700	gi|157119361	*cytochrome P450 49a1*	0.769	0.022
comp44013	gi|170049356	*cytochrome P450 12b1*	0.772	0.004
comp30097	gi|916344537	*cytochrome P450 6BQ37*	−0.852	0.038
comp34369	gi|109603635	*cytochrome P450 9e2-like*	−1.506	0.003
comp36247	gi|906471773	*Cytochrome P450 18a1*	−0.917	0.006

### Quantitative real-time PCR validation of P450s

**Figure 3:**
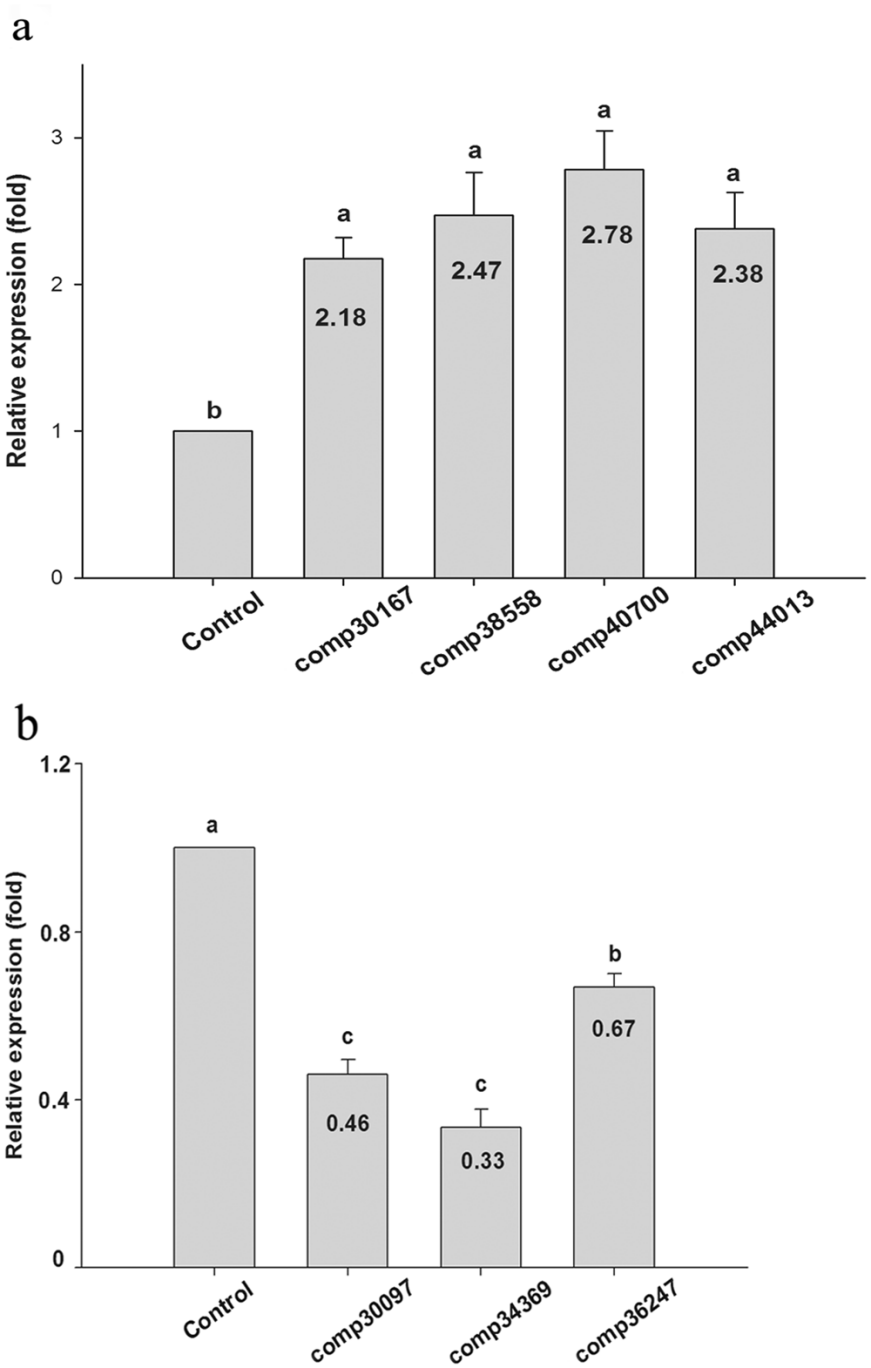
Relative expression levels of seven P450 unigenes in imidacloprid-treated *B. odoriphaga*. (**a**) Four upregulated P450s unigenes; (**b**) Three downregulated P450 unigenes. The transcription levels of the seven P450s unigenes were determined by quantitative real time PCR. Each bar indicates the mean of three biological replicates (±*SE*). 18 s was used as a reference gene. The different letters on the bars represent significant differences between treatment and control group based on Duncan’s multiple comparison test (*P* < 0.05). Numbers in each bar represent fold change compared with the control.

qRT-PCR was conducted to verify the expression level of seven P450 DEGs (4 upregulated and 3 downregulated) between the treatment group and the control group after imidacloprid treatment. The number of unigenes in each gene expression profile based on qRT-PCR (Fig. 3a,b) was similar to the RNA-Seq DEG gene expression data (Fig. S4).

### Functional analysis of P450s by RNAi

**Figure 4:**
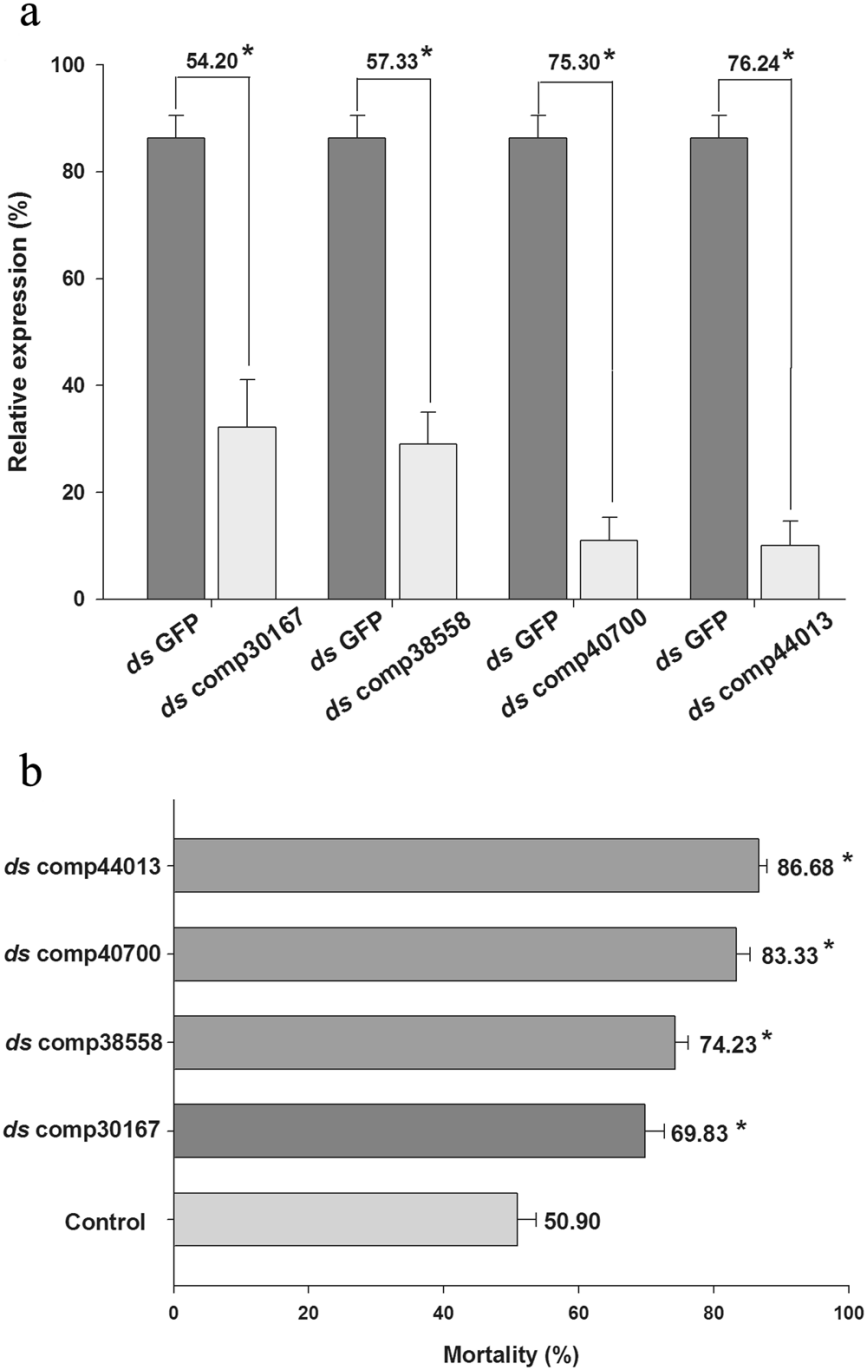
The *ds*RNA-mediated suppression of P450s transcript expression in *B. odoriphaga* fed on the artificial diet with *ds*RNA. The control group was fed with *ds*GFP. (**a**) Repressions of the transcripts of four P450s genes after larvae were fed an artificial diet with *ds*RNA for 48 h. The transcript levels of four P450s genes were examined using qRT-PCR, and 18 s was selected as a reference gene. (**b**) Insecticide bioassays were conducted 48 h after the uptake of P450 *ds*RNA by the standard contact and stomach bioassay method. Mortality was assessed 48 h after the insecticide treatments. Results are mean ± *SE* of three biological replications (n = 3). The asterisks on the bars indicate significant differences between the control and treatment group after P450s *ds*RNA uptake (Student’s *t*-test, *P* < 0.05).

After feeding *B. odoriphaga* larvae individual P450 *ds*RNAs, the relative expression levels of mRNA were examined to investigate the knockdown efficiency of P450 gene expression. After feeding larvae the specific P450 *ds*RNAs, the expression of the target P450s gene decreased significantly compared to the control (Fig. 4a), indicating an effective silencing of P450s by RNAi in *B. odoriphaga*. Furthermore, after feeding larvae P450 *ds*RNAs, the mortality of *B. odoriphaga* larvae caused by imidacloprid increased significantly, with increases ranging from 18.93 to 35.78%, when treated with an LC_50_ dose of imidacloprid, Fig. 4b.

## Discussion

To discover and annotate the genes involved in imidacloprid detoxification, we used *de novo* assembly and analyzed differentially expressed genes in *B. odoriphaga* after imidacloprid treatment. This approach was necessary because *B. odoriphaga* does not have a reference genome. In this study, an Illumina platform was used to sequence the *B. odoriphaga* transcriptome. We identified 43,453 unigenes, and 19,442 of these unigenes were found to be above the assigned cutoff (e < 1e-5) after BLAST analysis using the NR database. The results were similar to previous RNA-seq studies on *B. odoriphaga* by Gao^26^, in which 47,578 unigenes were assembled using *de novo* sequencing. This subgroup of unigenes was further annotated using the GO and KEGG databases. These unigenes from transcriptome sequencing after imidacloprid treatment could provide useful information at the genomic level for controlling *B. odoriphaga* and other dipteran insects.

To discover the genes related to imidacloprid detoxification in *B. odoriphaga*, the functions of DEGs were annotated from the GO and KEGG databases according to the annotated information of all unigenes of *B. odoriphaga*. By searching the GenBank database, the four up-regulated unigene comp30167, 38558, 40700 and 44013 were annotated similar with *CYP9b2* in *Culex quinquefasciatus*, P450s gene in *Anopheles darlingi* genome, *CYP49a1* in *Aedes aegypti* and *CYP12b1* in *Culex quinquefasciatus*, respectively. While it had been reported that *CYP9b2* in *Drosophila melanogaster* was related to toxin resistance^29^, *CYP49a1* expressed in *Bombyx morito* was involved in phoxim metabolism^30^ and *CYP12b1* as a mitochondrial P450 may be related to biological process in *Drosophila acanthoptera*^31^. So far, no any imidaclodprid detoxification related annotation could be found for the four up-regulated P450 unigenes. Thus, in order to give an insight into the gene functions, we conducted qRT-PCR to verify the response of these DEGs after imidacloprid treatment. Moreover, RNAi was used for characterization of the roles of upregultaed P450 unigenes in detoxification of imidacloprid.

In addition, among the *B. odoriphaga* transcripts related to imidacloprid detoxification, four P450 differentially expressed transcripts were upregulated and three P450 transcripts were downregulated. In insects, more than half of all P450 genes belong to only two subfamilies, CYP 4 and CYP 6^32^. In our study, one differentially expressed P450s transcripts belonged to CYP 6 and two belonged to the CYP 9 clade. Genes in the CYP 6 subfamily are involved in imidacloprid detoxification metabolism in *Bemisia tabaci*, *Myzus persicae*, *Nilaparvata lugens*, and *Musca domestica*^33–36^, while the CYP 9 subfamily genes are related to the oxidative metabolism of insecticides^18^.

To evaluate the validity of Illumina analysis after imidacloprid treatment, a total of seven differentially expressed unigenes, including four upregulated and three downregulated P450 transcripts, were selected to test expression changes by qRT-PCR between the imidacloprid treatment group and the control group. The variation in expression of these unigenes is shown in Fig. 3a.b. The qRT-PCR results of these seven P450 DEGs related to imidacloprid detoxification were identical to those obtained by DEG expression profiling.

Post-transcriptional gene silencing by RNA interference (RNAi) is a useful tool for examining the functions of individual genes^37^. As the reported imidacloprid metabolism resistance was mainly related with up-regulate of P450s gene^38^. The functions of the 4 up-regulated P450s unigenes in imidacloprid metabolism resistance were further studied by RNAi, while the roles of down-regulated genes need to be examined in other work. Oral delivery mediated RNAi method has been applied to the study of gene function, including in insecticide detoxification metabolism genes^39–41^. Then, by using a feeding based RNAi technique, we were able to reduce the four P450s transcripts levels by 54.20 to 76.24% in larvae fed on a diet containing P450s *ds*RNA, compared to larvae fed on a diet containing GFP *ds*RNA. Suppression of the P450 transcripts in the larvae fed on P450s *ds*RNA resulted in a significant increase of imidacloprid sensitivity in *B. odoriphaga* larvae. This indicated that these four up-regulated P450 unigenes are involved in imidacloprid detoxification in *B. odoriphaga*. In addition, this finding also enriched the growing body of literature showing that the RNAi technique can be applied in *B. odoriphaga*. The low level of detoxification enzyme gene expression and elevated insecticide sensitivity has been widely found in many other kinds of insects after RNAi^42–45^. Overall, oral delivery mediated RNAi not only suggested that ingestion of *ds*RNA through an artificial diet could be exploited for functional genomic studies in *B. odoriphaga*, but also indicated that P450s can be considered a major control target for delaying resistance to neonicotinoid insecticides in *B. odoriphaga*. The full length and expression character of these upregulated P450 transcripts need to be further studied.

In conclusion, RNA-Seq techniques have revolutionized our understanding of molecular genetics in insecticide resistance. In this study, RNA-Seq was performed to obtain several differentially expressed P450 unigenes related to imidacloprid detoxification in *B. odoriphaga* after imidacloprid treatment. As expected, four upregulated and three downregulated P450 unigenes were found. qRT-PCR expression results were consistent with RNA-Seq data. Furthermore, gene silencing followed by an insecticide bioassay was applied to understand the role of the four upregulated P450s transcripts in imidacloprid detoxification, which indicated that these upregulated P450 transcripts played an important role in the detoxification of imidacloprid in *B. odoriphaga*. Information from the transcriptome, DEG analysis, and gene function experiment provide a genetic basis for investigating imidacloprid detoxification P450 genes in *B. odoriphaga*.

## Materials and Methods

### Insect material

The population of *B. odoriphaga* used in this study was obtained from the Key Laboratory of Pesticide Toxicology & Application Technique of Shandong Province, College of Plant Protection, Shandong Agricultural University. This strain has been maintained in the laboratory without exposure to any insecticides since 2013. They were maintained at 25 ± 1 °C and 60–70% relative humidity with a photoperiod of 16:8 h (L:D).

### Bioassay

Bioassays were conducted on newly emerged 4th instar larvae of *B. odoriphaga* using a standard contact and stomach bioassay method^46^. Briefly, three fresh Chinese chive pseudo stems and *B. odoriphaga* larvae were dipped into insecticide or control solutions for 15 s with gentle agitation. Then, the larvae and stems were transferred to a 9-well tissue-culture plate containing 2% agar covered with filter paper. Three replicates were conducted with at least 20 larvae for each replicate. Distilled water containing 0.1% (v/v) Triton X-100 and 1% acetone was used as a control. Mortality was assessed after 24 h. Larvae were considered dead if they were unable to move when touched by a moist brush. Probit analysis was used to calculate the median lethal concentration (LC_50_) with 95% confidence intervals (SPSS 19.0). The treatment group was newly emerged 4th instar larvae exposed to an LC_50_ concentration of imidacloprid for 24 h, and the control group was treated with the control solution. Two replicates were used in the treatment group and in the control group, and four samples in total were used for transcriptomic analysis.

### RNA isolation and quality controls

Total RNA was extracted from 20 larvae in each sample using TRIzol reagent (Ambion, USA) according to the manufacturer’s protocol. The quality, quantity, and integrity of each RNA sample were assessed using two spectrophotometers: the Nanodrop (Thermo Scientific, USA) and the Aglient 2100 (Life Tech, USA). RNA samples were only used if they had a 260:230 ratio from 2.0 to 2.5, a 260:280 ratio from 1.9 to 2.1, and an RNA integrity number (RIN) higher than 8.0.

### Illumina sequencing and *de novo* assembly

According to the Illumina manufacturer’s instructions, mRNA was enriched from DNase I-treated total RNA using the oligo (dT) magnetic beads, and cDNA was reverse-transcribed using the random hexamer primer. After purification with magnetic beads, cDNA was ligated at the 39-end with adenine and sequencing adaptors, followed by PCR amplification to create a cDNA library.

The cDNA library was sequenced on an Illumina sequencing platform (Hiseq. 2500, Illumina, USA). After removal of low-quality reads containing primer/adaptor sequences and trimming of read lengths using NGS QC Toolkit (v2.3.3), high-quality reads considered as clean data with an identity value of 95% and a coverage length of 125 bp were assembled *de novo* using Trinity (vesion:trinityrnaseq_r20131110) software and clustered using the TGICL to generate unigenes (Shanghai Oebiotech Co., Ltd., China)^47–49^.

### Sequence clustering and functional categorization of unigenes

Unigene annotation of *B. odoriphaga* in the treatment and control groups was performed by searching the GenBank database with the BLASTX algorithm (http://www.ncbi.nlm.nih.gov/). NR (ftp://ftp.ncbi.nih.gov/blast/db/), GO (http://www.geneontology.org/), SWISSPROT (http://www.uniprot.org/downloads), KOG (http://www.ncbi.nlm.nih.gov/COG/), and KEGG (http://www.genome.jp/kegg/) annotations of the unigenes were determined using BLAST software.

### Differentially expressed genes after imidacloprid treatment in *B. odoriphaga*

Reads sequenced from each sample of *B. odoriphaga* were aligned with the unigene library by Bowtie (http://bowtie-bio.sourceforge.net/index.shtml). To obtain relative expression levels in each sample, fragments per kilobase of transcript per million mapped reads (FPKM) in each sample were counted. The differential gene expression analysis was then performed using DESeq (http://www.bioconductor.org/packages/release/bioc/html/DESeq.html) for the treatment group and the control group. A gene was considered to be differentially expressed when results from the above tests were all significant at a level of *P* ≤ 0.05. The GO and KEGG annotations were performed using BLAST software for the differential expression of genes.

### Reverse transcription (RT-PCR) and quantitative real-time PCR (qRT-PCR) validation

Approximately 20 *B. odoriphaga* larvae were collected in an Eppendorf tube. Total RNA was extracted using RNA-Solv^@^ Reagent (E.Z.N.A., USA) according to the manufacturer’s instructions. First-strand cDNA was synthesized from 1 μg of the total RNA using a PrimeScript RT-reagent Kit with gDNA Eraser (TaKaRa Biotechnology, China). Three independent RNA preparations representing three biological replicates were used for cDNA synthesis. The expression levels of P450 genes were quantified by qRT-PCR using a Biosystems 7500 Real-time PCR System (Applied Biosystems Inc, Foster City, USA) following the manufacturer’s protocols with the SYBR® Premix Ex Taq™ II kit (TaKaRa). Each 20 μL reaction contained 10 μL SYBR Green, 0.4 μL of each primer, 0.4 μL ROX, 1 μL cDNA template, and double distilled water. The cycling parameters consisted of an initial step at 95 °C for 30 s, followed by 40 cycles of 95 °C for 5 s and 60 °C for 34 s. The relative expression of each P450 gene was calculated according to the 2^−ΔΔCT^ method^50^. Difference analysis was performed by using Duncan’s multiple comparison test with SPSS software in transcriptome verification experiment. Each qRT-PCR experiment consisted of three independent biological replicates, each with three technical replicates.

### RNAi and bioassays with imidacloprid after RNAi

Upregulated P450s transcripts were selected as candidates from differentially expressed gene (DEG) analysis. Then, *ds*RNAs were synthesized from purified PCR products using the Transcript Aid T7 High Yield Transcription Kit (Thermo Fisher Scientific, Waltham, MA) according to the manufacturer’s instructions. PCR was performed using gene-specific primers containing T7 polymerase sites. All primer sets are listed in Table S1. PCR was performed with cycling parameters starting with 94 °C for 3 min, followed by 35 cycles of 94 °C for 30 s, 56 °C for 30 s and 72 °C for 45 s, with a final extension at 72 °C for 10 min. Template DNA and single-stranded RNA were removed from the transcription reaction with DNase and RNase treatments, respectively. The *ds*RNA was purified using the phenol (pH 4.7): chloroform extraction and ethanol precipitation methods and eluted in diethyl pyrocarbonate (DEPC)-treated nuclease-free water. The *ds*RNA concentrations were measured using a Nanodrop spectrophotometer. DEPC-treated nuclease-free water was used as the negative control. Difference analysis was performed by using student *t* tests with SPSS software. A value of *P* < 0.05 was considered significant. The same for the following experiment.

To evaluate the functional role of specific P450s genes, *B. odoriphaga* 4^th^ instar larvae fed with *ds*RNA-P450s at a concentration of 30 μg/g artificial diet and with an interval of 48 h post-feeding were used for qRT-PCR and bioassay. Primers used in this study are shown in Supplementary Table S1.

The LC_50_ dose of imidacloprid was applied for bioassays of the *B. odoriphaga* larvae after RNAi. The 4th instar larvae of *B. odoriphaga* were treated with different P450 *ds*RNA for 48 h. The control group was fed with an artificial diet mixed with GFP *ds*RNA. Bioassays were conducted as described in bioassay section.

## Electronic supplementary material

Supplementary Information
