# Supplementary material for: Transcriptome analysis and identification of P450 genes relevant to imidacloprid detoxification in Bradysia odoriphaga
Source: Sci Rep. 2018 Feb 7;8:2564. doi: 10.1038/s41598-018-20981-2 (PMC5803201; doi:10.1038/s41598-018-20981-2)
Supplement: Supplementary file 1 — Supplementary Information [file 41598_2018_20981_MOESM1_ESM.pdf]

Transcriptome analysis and identification of P450 genes relevant to imidacloprid detoxification in *Bradysia odoriphaga*

Chengyu Chen[1], Cuicui Wang[1], Ying Liu[1], Xueyan Shi[1*], Xiwu Gao[1]

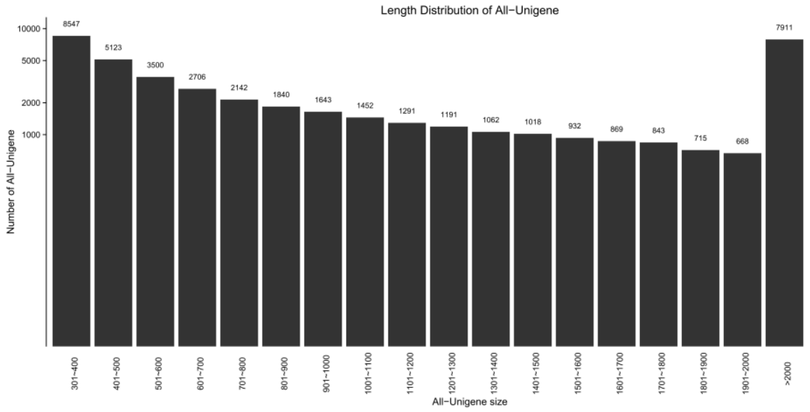

Figure S1 Length distribution of *B. odoriphaga* unigenes. The x-axis indicates the length distribution of all unigenes and the y-axis indicates unigene number.

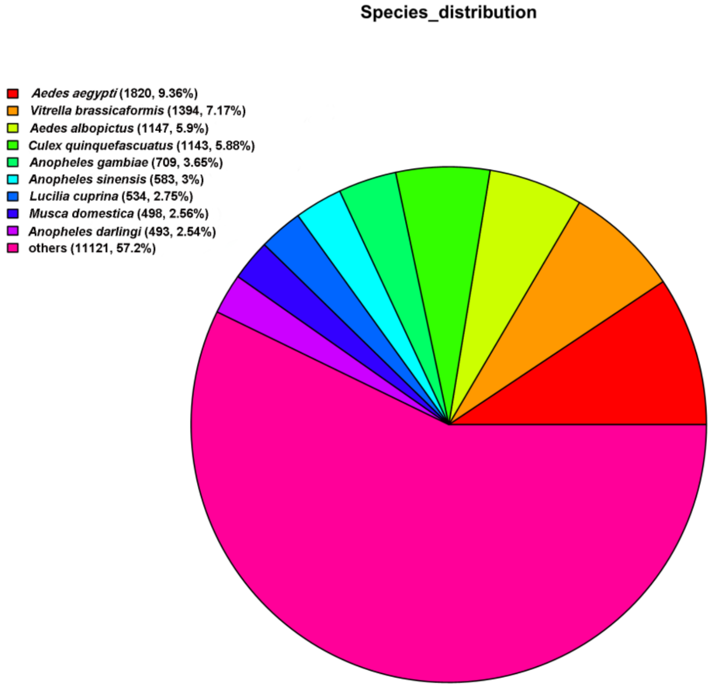

Figure S2 Match result of non-redundant protein (NR) homologous species distribution. The number means the matched unigenes with the corresponding homologous species for all unigenes

of *Bradysia odoriphaga*.

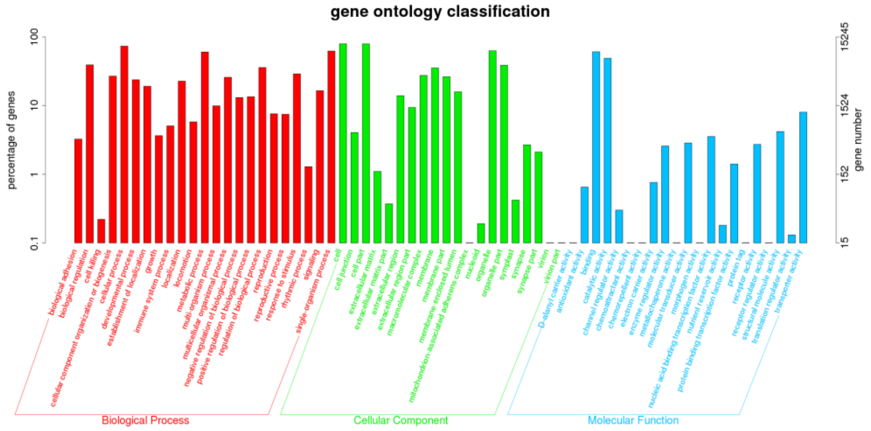

Figure S3. Number of *Bradysia odoriphaga* unigenes in each functional category. Unigenes were classified into different functional groups based on the Gene Ontology (GO) data library within the groups: biological process (red), cellular component (green), or molecular function (blue). The x-axis indicates the sub-categories and the y-axis indicates the percentage (left) and number (right) of all unigene.

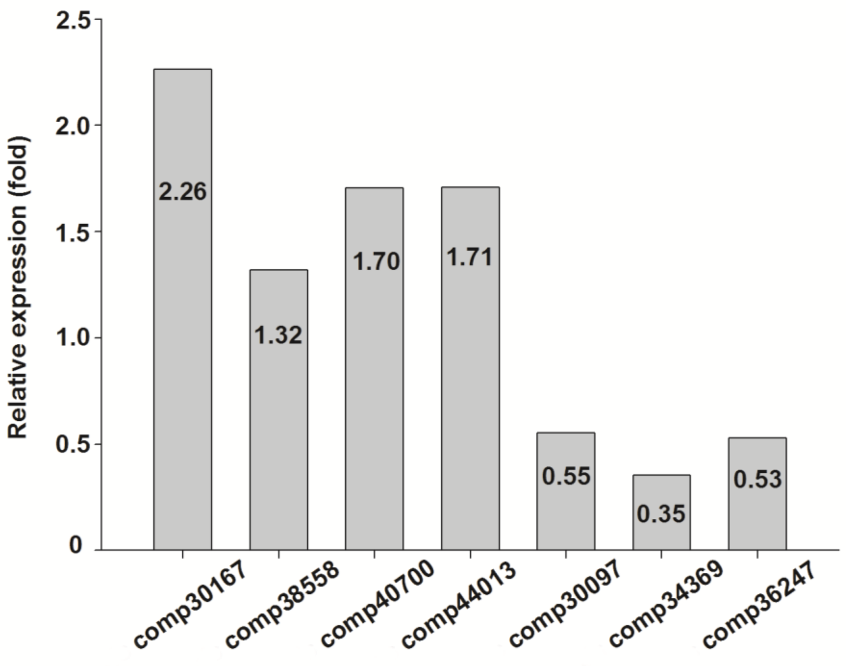

Figure S4 DEG analysis of seven differentially expressed P450 transcripts used in our study. Numbers in each bar represent the fold change of 7 P450s unigenes, normalized to control, respectively.

Table S1. The specific primers used in qRT-PCR and RNAi

Unigene ID	Up-down	Primer used	Primers sequence (5'-3')
comp30167	up	qRT-PCR	F-GCCACACTCAGTCCAACATTC
			R-GACATCGTTCGTATAGCGTG
		RNAi	F-taatacgactcactatagggACATTTGTCGGAAAAGGCTG
			R- taatacgactcactatagggTGCCAGCAATTCATTGTCAT
comp38558	up	qRT-PCR	F-GGCGTGATATGAGAGCTACCC
			R-GCCGCTGACGAAATGACATC
		RNAi	F-taatacgactcactatagggGCGGCTTTTGGTTACAACAT
			R-taatacgactcactatagggCGTTCCTGAATGTCAGGGTT
comp40700	up	qRT-PCR	F-GCCGTTAACGTTCTACCAGC
			R-CCAATAATCGGTGCAAATCG
		RNAi	F-taatacgactcactatagggTACGCGATTGGGTTGTGTAA
			R-taatacgactcactatagggCTCTCAGAAACGGCATCCTC
comp44013	up	qRT-PCR	F-CATTCGTCGCACTACTCCTC
			R-GCCAATGGGAACGAGCGAAC
		RNAi	F-taatacgactcactatagggCGAGCAATAGATCGGGACAT
			R-taatacgactcactatagggGGTGACACATTTTGCACCTG
comp30097	down	qRT-PCR	F- GAGTCGAGGATGAAGTCTC
			R-GTTAGTGATGGTAAGAATC
comp34369	down	qRT-PCR	F- TGTGCTCTTCCGATCTATC
			R-GTAGAAGGCGTAAGCTATG
comp36247	down	qRT-PCR	F- CTGAGATTGTTCGGAATGAC
			R-CAGCCACCGAATAAATGGGAC
18s		qRT-PCR	F- CTAACCATAAACGATGCCAG
			R- ACTCCACGAACTAAGAACGG
